# Editorial: Imaging brain network and brain energy metabolism impairments in brain disorders

**DOI:** 10.3389/fnmol.2025.1676946

**Published:** 2025-08-14

**Authors:** Yuhei Takado, Mor Mishkovsky, Tomokazu Tsurugizawa

**Affiliations:** ^1^Institute for Quantum Life Science, National Institutes for Quantum Science and Technology (QST), Chiba, Japan; ^2^Laboratory for Functional and Metabolic Imaging (LIFMET), Ecole Polytechnique Fédérale de Lausanne (EPFL), Lausanne, Switzerland; ^3^National Institute of Advanced Industrial Science and Technology (AIST), Tsukuba, Japan

**Keywords:** brain energy metabolism, functional connectivity, magnetic resonance spectroscopy, resting-state fMRI, astrocyte-neuron interactions, multimodal imaging

Brain disorders often emerge at the intersection of two fundamental dysfunctions: disrupted functional networks and impaired energy metabolism. While magnetic resonance spectroscopy (MRS) and resting-state functional MRI (rsfMRI) have traditionally provided separate windows into these domains, this Research Topic was launched with the hypothesis that functional and metabolic networks are not isolated; rather, they are tightly interconnected. Our aim was to bring together diverse imaging modalities and analytical frameworks to explore how metabolic disorders and network dysfunctions co-evolve in various neurodegenerative and neurodevelopmental diseases.

The concept of the astrocyte–neuron lactate shuttle (ANLS), originally proposed by Pellerin and Magistretti (1994), provides a mechanistic framework for understanding how neuronal activity is metabolically coupled to astrocyte-mediated glycolysis. These insights underscore the inseparability of functional and metabolic networks in the brain. Electrophysiological studies have demonstrated that spontaneous BOLD fluctuations in the default mode network (DMN) are closely tied to low-frequency neuronal activity, particularly local field potentials (LFPs) (Logothetis et al., 2001; He et al., 2008; Schölvinck et al., 2010). At the same time, positron emission tomography (PET) studies have shown that DMN regions exhibit elevated aerobic glycolysis (Vaishnavi et al., 2010), a metabolic phenotype largely attributed to astrocytic function. These complementary findings suggest that the DMN arises from a coordinated interplay between neuronal and astrocytic activity, rather than from neural activity alone. Therefore, it is essential to approach brain network and brain energy metabolism as interconnected systems, as exemplified by the contributions in this Research Topic.

The articles included in this Research Topic reflect a wide conceptual and methodological range, spanning basic neurochemistry, advanced imaging techniques, translational animal models, and human clinical populations. Though no single study combined MRS and rsfMRI directly, the ensemble provides a multilayered view of how brain networks and energy states are interconnected.

Watanabe et al. proposed the “ATP Supply–Demand Mismatch Model” to explain Parkinson's disease (PD) pathogenesis. This model posits that disruptions in multiple energy-generating pathways—including glycolysis, the pentose phosphate pathway, and purine metabolism—lead to ATP depletion. The resulting energy deficit impairs protein degradation systems such as the autophagy-lysosomal and ubiquitin-proteasome pathways, causing α-synuclein aggregation and neuronal network breakdown. The article also emphasizes the vulnerability of cortical hubs and the massive axonal arborizations in the striatum to energy failure, thus linking network-level disintegration to both motor and non-motor symptoms of PD (Watanabe et al.).

In a related effort, Duarte explored how hippocampal glutamate and NAA levels measured by high-field ^1^H MRS in rats correlate with spontaneous alternation behavior, suggesting that even subtle metabolic variation predicts cognitive performance.

Hyperpolarized ^13^C MRSI in awake mice, as demonstrated by Ono et al., allowed dynamic assessment of brain metabolism under physiological conditions. They showed that anesthesia suppresses mitochondrial flux and that aging selectively impairs hippocampal oxidative metabolism, revealing the sensitivity of metabolic imaging to both cerebral conscious state and age (Ono et al.).

On the network side, Ouchi et al. examined age-related network changes in macaque monkeys using structural and functional MRI. They observed a decrease in structural connectivity and a paradoxical increase in functional indices with age, providing cross-species insights into aging-related network plasticity (Ouchi et al.).

Within the broader study of human brain disorders, Xue et al. investigated early-stage coal workers' pneumoconiosis (CWP), specifically assessing differences between patients with and without a history of alcoholism. Their rsfMRI analysis revealed both Amplitude of Low-Frequency Fluctuation (ALFF) and functional connectivity abnormalities in key prefrontal and posterior regions, some of which correlated with clinical markers such as PaO_2_ and forced vital capacity (FVC), suggesting systemic effects on brain function (Xue et al.).

Das et al. focused on white matter and subcortical gray matter network alterations in temporal lobe epilepsy. By analyzing both static and dynamic functional connectivity, they uncovered reductions in integration and variability, highlighting the importance of deep brain structures in epileptogenic networks (Das et al.).

Beyond biological findings, methodological innovation also played a key role in this Research Topic. In addition to mechanistic and disease-focused studies, this Research Topic also features articles that provide resources for MRS/MRSI data analysis and processing pipelines, which are crucial for reproducible spectroscopy-based investigations. Xiao et al. introduced MRSpecLAB, a user-friendly, open-access software platform for MRS and MRSI data analysis. MRSpecLAB features a graphical pipeline editor, supports multiple nuclei (^1^H, ^31^P, ^13^C), and allows both standardized and customizable processing workflows. By integrating LCModel and enabling batch analysis and plug-and-play modules, this tool reduces the technical barriers for non-experts and fosters reproducible MRS studies across neuroscience and clinical domains.

In summary, these studies highlight the necessity of integrating metabolic and functional imaging perspectives to fully understand brain network organization and dysfunction. This Research Topic, by bridging MRS, rsfMRI, and metabolic theory, represents a timely contribution to this evolving field. We hope these contributions stimulate further dialogue, inspire novel hypotheses, and support future development of multimodal imaging biomarkers for neurological and psychiatric disorders.
